# μVEMP: A Portable Interface to Record Vestibular Evoked Myogenic Potentials (VEMPs) With a Smart Phone or Tablet

**DOI:** 10.3389/fneur.2018.00543

**Published:** 2018-07-05

**Authors:** Hamish G. MacDougall, John Holden, Sally M. Rosengren, Elodie Chiarovano

**Affiliations:** ^1^School of Psychology, University of Sydney, Sydney, NSW, Australia; ^2^Neurology Department, Royal Prince Alfred Hospital, Sydney, NSW, Australia; ^3^Central Clinical School, University of Sydney, Sydney, NSW, Australia

**Keywords:** ocular VEMPs, cervical VEMPs, oVEMPs, cVEMPs, utricle, saccule, otoliths, vHIT

## Abstract

**Background:** Cervical VEMPs and ocular VEMPs are tests for evaluating otolith function in clinical practice. We developed a simple, portable and affordable device to record VEMP responses on patients, named μVEMP. Our aim was to validate and field test the new μVEMP device.

**Methods:** We recorded cervical VEMPs and ocular VEMPs in response to bone conducted vibration using taps tendon hammer to the forehead (Fz) and to air conducted sounds using clicks. We simultaneously recorded VEMP responses (same subject, same electrode, same stimuli) in three healthy volunteers (2 females, age range: 29–57 years) with the μVEMP device and with a standard research grade commercial (CED) system used in clinics. We also used the μVEMP device to record VEMP responses from six patients (6 females, age mean±SD: 50.3 ± 20.8 years) with classical peripheral audio-vestibular diseases (unilateral vestibular neuritis, unilateral neurectomy, bilateral vestibular loss, unilateral superior canal dehiscence, unilateral otosclerosis).

**Results:** The first part of this paper compared the devices using simultaneous recordings. The average of the concordance correlation coefficient was rc = 0.997 ± 0.003 showing a strong similarity between the measures. VEMP responses recorded with the μVEMP device on patients with audio-vestibular diseases were similar to those typically found in the literature.

**Conclusions:** We developed, validated and field tested a new device to record ocular and cervical VEMPs in response to sound and vibration.This new device is portable (powered by a phone or tablet) with pocket-size dimensions (105 × 66 × 27 mm) and light weight (150 g). Although further studies and normative data are required, our μVEMP device is simpler (easier to use) and potentially more accessible than standard, commercially available equipment.

## Introduction

Vestibular-evoked myogenic potentials (VEMPs) are commonly used for clinical neurophysiological assessment of the vestibular system for patients with complaints of dizziness, vertigo, oscillopsia, imbalance, and falls. Cervical VEMPs (cVEMPs) and ocular VEMPs (oVEMPs) are tests for evaluating otolith function for research and in clinical practice (1, 2).

VEMPs are short latency reflexes elicited in response to vestibular stimulation: air-conducted sound (ACS) delivered via the external auditory canal, or bone-conducted vibration (BCV) using tendon hammer taps or a clinical vibrator on the head (3). In clinics, VEMPs are most commonly recorded using surface electromyography (EMG). Vestibular stimulation causes activation of afferent vestibular neurons. In oVEMPs, this neuronal activation induces a short latency crossed-reflex activation of the extra-ocular inferior oblique muscle. These myogenic potentials can be measured by surface electrodes positioned beneath the contralateral eye (4–7). In cVEMPs, myogenic potentials are measured from the ipsilateral sternocleidomastoid (SCM) muscle (8).

VEMPs have enabled the evaluation of the function of the otolithic organs. cVEMPs have been utilized for assessing the sacculo-collic reflex pathway and reflect the uncrossed inhibitory potentials originating from the saccule and the inferior vestibular nerve (8–10). oVEMPs evaluate the vestibulo-ocular reflex pathway and have been demonstrated to reflect the crossed excitatory potentials originating primarily from the utricle and the superior vestibular nerve (7).

Since the early VEMP paper published in 1994 (8), literature on VEMPs has increased and hundreds of papers have now been published. The use of VEMPs as a vestibular test providing information about otolithic function has gained a place in the standard vestibular test battery. VEMPs provide rapid and clinically useful information about the vestibular apparatus [for review see (11, 12)].

In this project, we developed, validated and field tested a new device to record VEMP responses on patients. This new device is mobile and portable because of its low weight and small dimensions. The usual laptop or desktop computer has been replaced by a smart phone or inexpensive tablet. The bio-amplifiers and data acquisition interface have been miniaturized to a pocket size device (105 × 66 × 27 mm, 150 g). We call this device μVEMP (“micro-VEMP” or “you-VEMP”) in reference to its small size, the typical response units (microvolts), and the focus on easy operation by “you.”

Our aim was to validate the data from the new μVEMP device and to evaluate its usability in the clinical environment on patients. To this end, we simultaneously recorded VEMPs on healthy subjects with the μVEMP device and with a commercially available standard system used at the Royal Prince Alfred teaching and research hospital. The first part of this paper shows the comparison between both devices. We also recorded VEMP responses from patients with classical peripheral audio-vestibular diseases.

## Methods

The study was approved by the local ethics committee (Adventist HealthCare HREC 2017-021) and participants gave informed written consent according to the Declaration of Helsinki.

### μVEMP device description

**Figure 1:**
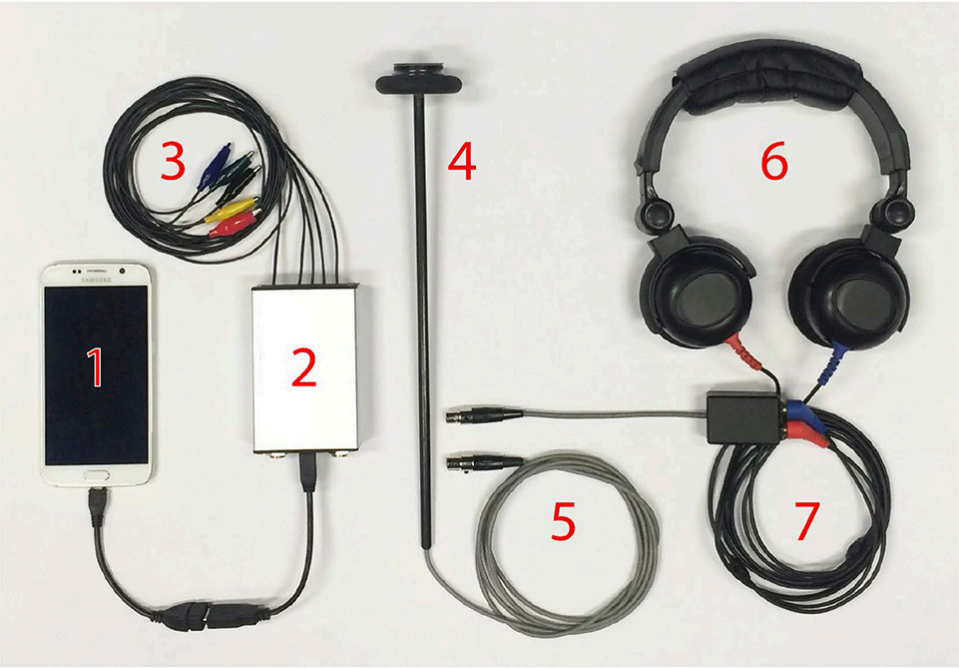
Picture of the new μVEMP device which consists of: (1) a smart phone (tablet, laptop or desktop computer); (2) a biopotential measurement device with five leads; (3) coaxial shielded cables, and “alligator” clips (for ECG electrodes); (4) a “smart” tendon hammer for bone-conducted vibration stimuli; (5) connection to generic peripheral ports; (6) audiometric headphones for air-conducted sound stimuli, and (7) a “smart” peripheral interface for auditory clicks.

A picture of the μVEMP device is given in Figure 1 and a schematic block diagram is given in Supplementary Material 1 (Figure S1).

Figure 1-1: A smart phone (Android OS), tablet (Windows 8 or 10), laptop or desktop computer is used for: user-interface, display, file storage, report generation, and networking. This connects to the μVEMP device using USB 2.0, USB OTG, Serial UART, Bluetooth or WIFI. In this study, VEMP responses were recorded with a Levono tablet running on Windows 8.

**Figure 2:**
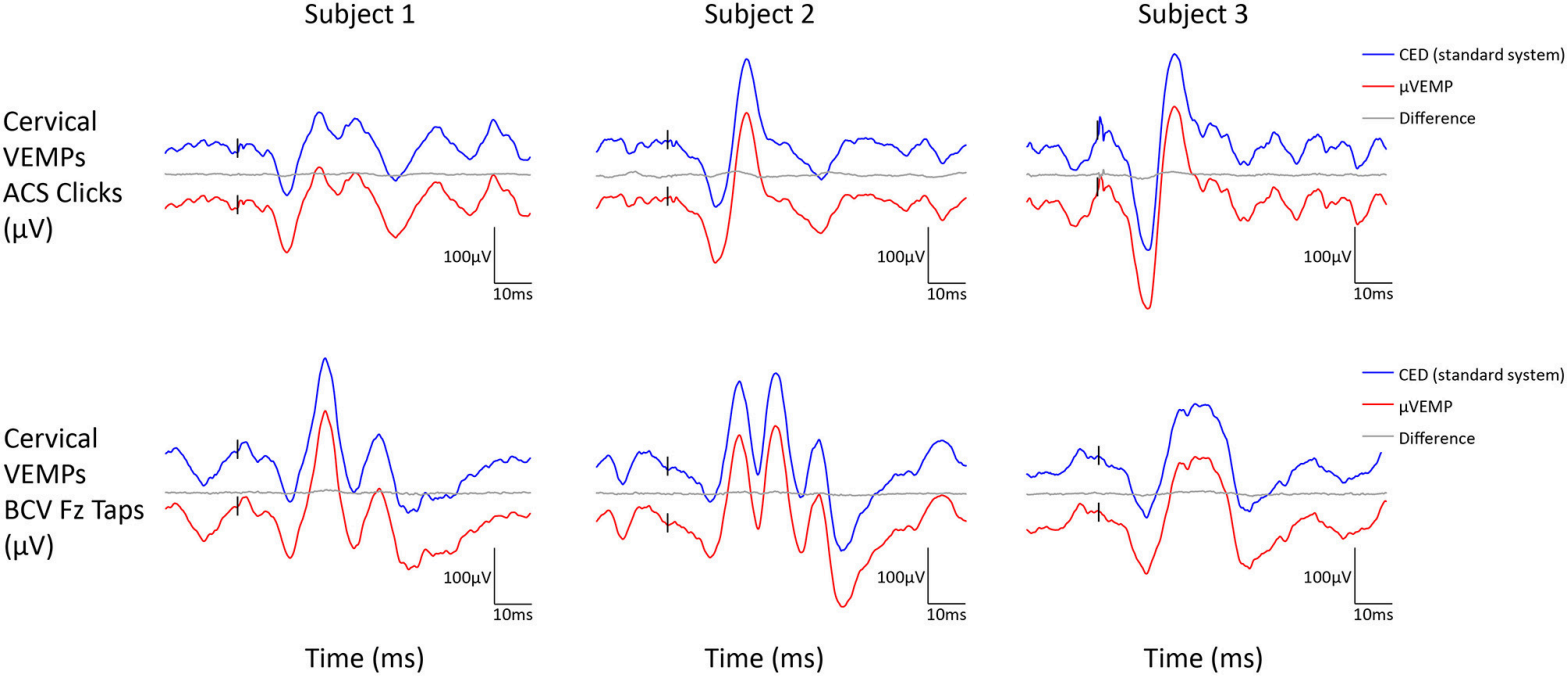
Cervical VEMP responses from the right ear recorded simultaneously with the μVEMP device (red curves) and the standard CED device (blue curves) from the three healthy subjects in response to ACS clicks and BCV Fz taps. Notice the two curves have been offset by 100 μV for clarity and the difference between the two curves is shown (gray curves).

Figure 1-2: The μVEMP biopotential measurement device contains 4–8 individual, differential input, 24-bit simultaneous-sampling analog-to-digital converters (ADCs) with programmable gain and low noise (< 1 μV), an ARM Cortex-M4 core Microcontroller with DSP and FPU running at 96 MHz, and medical grade electrical isolation rated at 5 kV.

**Figure 3:**
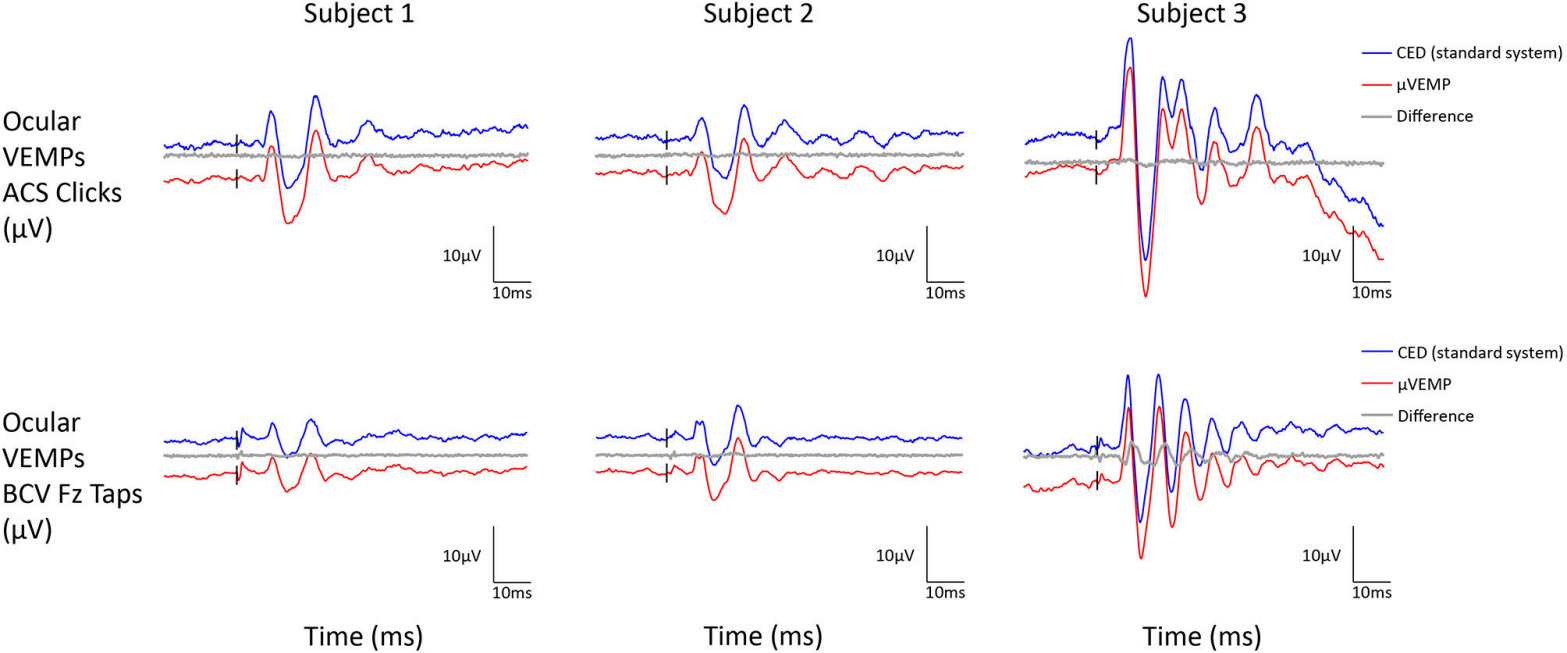
Ocular VEMP responses from the right ear recorded simultaneously with the μVEMP device (red curves) and the standard CED device (blue curves) from the three healthy subjects in response to ACS clicks and BCV Fz taps. Notice the two curves have been offset by 10 μV for clarity and the difference between the two curves is shown (gray curves).

Figure 1-3: Standard ECG electrodes (not shown) are connected to the μVEMP device using coaxial shielded cables and “alligator” clips.

Figure 1-4: BCV stimulus is delivered using a “smart” tendon hammer with integrated 3-axis accelerometer. An ARM Cortex-M0 Microcontroller calculates resultant accelerations (omnidirectional), provides TTL triggering with programmable thresholds and provides operator feedback by driving a RGB led to indicate low (blue; 4–10 g) medium (green; 10–16 g) or high (red; over 16 g) nominal tap intensities.

Figure 1-5: The hammer is connected to one of the μVEMP peripheral ports for power, serial communications, and TTL trigger/ready signals.

Figure 1-6: ACS stimulus is delivered by audiometric headphones (DD52, 10 Ω ± 1.0 Ω, Rated Power: 1,000 mW, Frequency response: 100 Hz−8 kHz, Sensitivity: 108 ± 3 dB SPL@1000 Hz @ 1 mW, Harmonic distortion:Below 1% @120 dB SPL, f = 1 kHz).

Figure 1-7: A “smart” peripheral interface generates auditory click signals (square wave pulses) for 105.0 dB LAeq. The interface contains an ARM Cortex-M0 Microcontroller which is programmed with variables such as pulse duration and stimulus repetition rate, nominally 0.1 ms and 5.1 Hz respectively.

The μVEMP device is modular with multiple generic communication ports to allow connection to various combinations of “smart” (containing microcontrollers) peripheral devices such as: the BCV Fz taps, the ACS click generator, a loop back tester, an isolated external triggering interface (TTL in and out), remote controllers, wireless transceivers, human interface devices, displays, and other equipment.

These 6-pin ports carry power for the “smart” peripheral device, bidirectional communications (serial UART), and TTL signals to indicate states such as “trigger” and “busy.” These generic communication ports provide for flexible system configuration and additional functionality in the future but also simplify operation for the user because the μVEMP device can recognize connected peripherals (for example to switch desired test type) and control them automatically.

Circuit designs, PCB layouts, CAD modeling, 3D printing, firmware and software development were all conducted in our laboratory. The intensity of the air conducted stimuli was calibrated using a class 1 sound level meter (Type 2250), a condenser microphone (Type 4144), and an artificial ear (Type 4152) all from Brüel & Kjær (DK-2850 Nærum Denmark).

### Simultaneous recordings

Simultaneous cVEMP and oVEMP recordings were performed on three healthy volunteers (2 females, age range: 29–57 years). The comparison device was a laboratory interface (Micro1401) and a bank of 1902 amplifiers coupled with Signal software (all from Cambridge Electronic Design Ltd (CED), Cambridge, United Kingdom).

The stimuli were ACS (clicks 0.1 ms, 105.0 dB LAeq, 5.1 Hz, ~100 repetitions) delivered to the right ear and BCV (taps, ~50 repetitions) applied to the forehead (Fz position). The stimuli were generated by the μVEMP device (for ACS clicks) and by the modified tendon hammer described above (for BCV Fz taps). The μVEMP device provided a TTL signal used to trigger the CED micro1401. In this way, the two machines could be synchronized: each tap with the hammer or each click with the headphone triggered data recording simultaneously on both devices.

cVEMPs were recorded from ECG surface electrodes (Cleartrace, Conmed Corp., Utica New York, USA) placed on the right, ipsilateral, SCM muscle. An active electrode was placed on the upper third part of the SCM muscle; a reference electrode was placed on the end of the scapula; and the “ground” was at the top of the sternum. oVEMPs were recorded from a pair of electrodes placed beneath the left, contralateral, eye. An active electrode was placed below the inferior eyeline; a reference electrode was placed below the active electrode; and the “ground” was at the top of the sternum. Negative potentials at the active electrodes were graphed as upward deflections.

Three cables were attached with alligator clips to each active and reference electrode. One cable provided data to the μVEMP device, and the other two provided data to two adjacent CED 1902 EMG amplifier channels with different filter settings. Two cables were attached to the ground electrode, one for each recording system. The main comparison was between the μVEMP data and the first CED channel (gain 1000), which were both recorded with filters switched off. The second CED channel had the usual clinical filters of 5 Hz−2 kHz and a gain of 3000. Data from this channel were used for development purposes and are not presented here. All channels recorded EMG with a sample rate of 4 kHz from 20 ms before to 80 ms post-stimulus. Data were exported from both systems as text files and analyzed offline. The unfiltered raw data for each frame (stimulus) were centered to zero by dividing each data point by the mean amplitude over the full 100 ms frame length, i.e., to remove the DC offset produced by the open filters. Frames (typically 100 for ACS clicks and 50 for BCV Fz taps) were then averaged to enable measurement of the response.

cVEMP responses consisted of two early waves: a first positive p13 wave and a second negative n23. oVEMP responses consisted of two early waves: a first negative n1 wave and a second positive p1. The latencies of these peaks were measured in ms from the stimulus onset. The peak to peak amplitude was calculated in microvolts (μV) from the unrectified EMG. For the cVEMPs only, amplitude was also expressed as a ratio of the background activity of the SCM muscle. EMG activity was full-wave rectified and averaged offline and measured during the 20 ms pre-stimulus period. Corrected amplitude was calculated as the peak to peak amplitude divided by the measure of muscle contraction. The evoked potential ratio (EPr) was calculated as a percentage by: (amplitude left–amplitude right)/(amplitude left + amplitude right)^*^100. An absolute value of the EP ratio greater than 30% was considered as abnormal (13, 14). A negative EP ratio indicated an amplitude smaller from the right ear. A positive EP ratio indicated an amplitude smaller from the left ear. For cVEMPs, the EP ratio was calculated with the corrected amplitude. For oVEMPs, the EP ratio was calculated with the peak to peak amplitude.

VEMPs from both devices were superimposed and the RMS error was calculated for both reflexes in response to ACS and BCV stimuli for the three volunteers (12 comparisons in total). The concordance correlation coefficient (rc) was also calculated.

### Patient recordings

cVEMPs and oVEMPs were recorded with the μVEMP device in six patients (6 females, age mean ± SD: 50.3 ± 2 0.8 years) with classical ear diseases. The age range and diagnosis of these patients are given in Table 1. VEMP stimuli used were the same as described above (ACS clicks and BCV Fz taps). The electrode montage was different than described above: cVEMPs and oVEMPs were recorded from the right and left ears. For cVEMPs, the active electrode was placed on the upper third part of both left and right SCM muscles; a reference electrode was placed on the top of the sternum; and the ground was on the forehead. For oVEMPs, the active electrode was placed below the inferior eyeline of both eyes; reference electrode was placed below the active electrode; and the ground was on the forehead.

**Table 1:** Age and diagnosis of the six patients suffering from classical audio-vestibular diseases.

**Patient**	**Age range (years)**	**Diagnosis**	**Side**
1	40–45	Healthy	–
2	30–35	Vestibular neuritis	Left
3	45–50	Neurectomy	Right
4	80–85	Vestibular loss (VL)	Bilateral
5	65–70	Superior canal dehiscence (SCD)	Right
6	25–30	Otosclerosis	Left

*These patients showed cervical and ocular VEMP responses consistent with those in the literature*.

## Results

### Simultaneous recordings

All three subjects had present cVEMPs and oVEMPs in response to ACS clicks and BCV Fz taps. Separated (and superimposed) curves recorded with the μVEMP device and the CED system are given in Figure 2 (and Figure S2) for cVEMPs and Figure 3 (and Figure S3) for oVEMPs for the three subjects. The traces from both systems were strikingly similar. The average across all six instances (two stimulations, three subjects) of the RMS error of the simultaneous records was 1.86 ± 0.54 (min-max: 1.25–2.76) for cVEMPs and 0.40 ± 0.33 (min-max: 0.20–1.06) for oVEMPs. The average of the concordance correlation coefficient was rc = 0.997 ± 0.003 showing a strong similarity between VEMPs recorded with both systems.

### Patient recordings

Peak to peak amplitudes (raw and corrected), latencies, and EP ratio for the six patients are given in Table 2 for cVEMPs and Table 3 for oVEMPs. The healthy subject had similar, normal cVEMPs, and oVEMPs from both ears. The patient with vestibular neuritis showed normal cVEMPs but absent oVEMPs from the affected ear. The neurectomy for schwannoma removal patient showed no responses in cVEMPs and oVEMPs to both stimulations from the affected ear. The bilateral vestibular loss (BVL) patient had absent cVEMPs or oVEMPs to both stimulations from both ears. The superior canal dehiscence (SCD) patient showed normal cVEMP amplitudes and increased oVEMP amplitudes from the affected ear to both stimulations. The otosclerosis patient showed no responses to ACS stimulation from the affected ear to both cVEMPs and oVEMPs and normal responses to BCV Fz taps stimulation.

**Table 2:** Cervical VEMP results for the six patients with audio-vestibular diseases.

**Cervical VEMPs**									
	**ACS clicks**								
	**Left ear**				**Right ear**				
	**PP Amp**	**PP Corrected**	**Latency p13**	**Latency n23**	**PP Amp**	**PP Corrected**	**Latency p13**	**Latency n23**	**EP ratio**
	**(**μ**V)**	**Amp**	**(ms)**	**(ms)**	**(**μ**V)**	**Amp**	**(ms)**	**(ms)**	**(%)**
Healthy	394	2.32	14.5	21.5	544	3.76	15.7	23.5	−23.0
Neuritis (left)	198	2.08	13.7	21.5	139	2.47	15.5	23.0	−8.50
Neurectomy (right)	529	3.61	14.5	21.7	0	–	–	–	100
Bilateral VL	0	–	–	–	0	–	–	–	NR
SCD (left)	379	2.70	15.5	23.7	446	2.23	14.2	22.5	10.1
Otosclerosis (left)	0	–	–	–	227	2.46	14.7	21.7	−100
**BCV Fz Taps**									
Healthy	254	2.71	13.5	20.0	279	2.92	13.5	20.2	−3.66
Neuritis (left)	70.0	1.09	15.0	19.7	78.0	1.25	14.7	20.0	−6.71
Neurectomy (right)	205	1.65	14.0	20.2	0	–	–	–	100
Bilateral VL	0				0	–	–	–	NR
SCD (left)	188	1.67	17.0	26.7	220	1.36	13.2	21.5	10.2
Otosclerosis (left)	125	2.14	13.7	22.5	126	2.31	14.2	22.7	−3.93

*This table shows peak to peak (PP) amplitudes, corrected amplitudes and latencies of the p13 and n23 waves for the left ear and the right ear as well as the EP ratio (comparison of the amplitude from both ears) in response to ACS clicks and BCV Fz taps. All the results are consistent with those in the literature. Note: in the EP ratio column, blue cells show an absence of response from one (pathological) ear; gray cells show an absence of response from both ears (due to bilateral disease); and white (transparent) cells show normal results (similar amplitudes from both ears)*.

**Table 3:** Ocular VEMP results for the six patients with audio-vestibular diseases.

**Ocular VEMPs**							
	**ACS clicks**						
	**Left ear**			**Right ear**			
	**PP Amp**	**Latency n1**	**Latency p1**	**PP Amp**	**Latency n1**	**Latency p1**	**EP ratio**
	**(**μ**V)**	**(ms)**	**(ms)**	**(**μ**V)**	**(ms)**	**(ms)**	**(%)**
Healthy	27.8	9.50	13.0	20.1	9.25	13.5	16.1
Neuritis (left)	0	–	–	13.1	9.00	13.7	−100
Neurectomy (right)	20.8	9.50	14.5	0	–	–	100
Bilateral VL	0	–	–	0	–	–	NR
SCD (left)	48.6	9.00	15.7	22.7	9.00	14.2	36.2
Otosclerosis (left)	0	–	–	31.4	9.25	13.0	−100
**BCV Fz taps**							
Healthy	24.7	9.00	13.5	26.0	9.00	14.0	−2.50
Neuritis (left)	0	–	–	22.1	8.75	14.7	−100
Neurectomy (right)	26.9	10.0	14.5	0	–	–	100
Bilateral VL	0	–	–	0	–	–	NR
SCD (left)	23.3	13.5	18.2	7.60	9.75	15.7	50.8
Otosclerosis (left)	43.3	9.00	13.2	42.4	9.50	14.0	1.05

*This table showed peak to peak (PP) amplitudes and latencies of the n1 and p1 waves for the left ear and the right ear as well as the EP ratio (comparison of the amplitude from both ears) in response to ACS clicks and BCV Fz taps. All the results are consistent with those in the literature. Note: in the EP ratio column, blue cells showed an absence of response from one (pathological) ear; red cells showed an asymmetry of the response (one ear is significantly larger than the other ear); gray cells showed an absence of response from both ears (due to bilateral disease); and white (transparent) cells showed normal results (similar amplitudes from both ears)*.

## Discussion

Since 2008 and the development of the first useable video head impulse test (vHIT) goggles, it is possible to assess the function of the six semicircular canals (15). Testing the remaining four otoliths end-organs still requires complicated and expensive equipment. In this project we developed a simple, portable and affordable VEMP device which we called the μVEMP (“micro-VEMP”). Similar to the strategy with the vHIT goggles, we employed recent advances in electronic component performance to miniaturize the VEMP device and simplified it by implementing only the minimum necessary features for effective testing. We recorded the traditional cVEMPs and oVEMPs in response to the simplest bone conducted vibration using taps and to air conducted sounds using clicks. Finally, the small size of the device makes it mobile and portable and so it could be used in new environments such as in emergency departments or outside the hospital.

To validate our new device, we simultaneously recorded VEMP responses in the same subjects, using the same electrodes and the same stimulation/recording parameters with the μVEMP and a standard commercially available device. Comparison showed very similar VEMP responses from both devices, validating our μVEMP device to record oVEMPs and cVEMPs. Responses recorded with the μVEMP device on patients with audio-vestibular diseases were the same as those typically reported in the literature for patients with unilateral vestibular neuritis (16, 17), unilateral neurectomy (14, 18), bilateral vestibular loss (19) and conductive hearing loss (20, 21). In a patient with superior canal dehiscence cVEMP and oVEMP amplitudes were enhanced, as expected (22–24), but we did not establish the threshold of responses. In order to interpret the results obtained on these patients, we used normative data for the EP ratio provided in the literature. Further studies are required to obtain normative data for our new μVEMP device.

One limitation of this intentionally simplified device is the range of stimuli available: VEMPs are induced with ACS clicks or BCV Fz taps only. Several other stimuli can be used to induce VEMP responses: ACS short tone bursts or BCV short tone bursts or square waves delivered with a mini-shaker (3). These other stimuli enable the vestibular system to be activated by stimuli with adjustable frequency content. It has been shown in the literature that different frequencies of the tone bursts may induce different responses in healthy subjects (25, 26) and in patients with SCD (27) or Meniere's Disease (28, 29). However, while the best stimulus frequency for inducing VEMP responses in healthy subjects may be around 500 Hz on average, many subjects or patients would better respond to an adjacent frequency [e.g., 250 or 1,000 Hz, (30)]. The optimal frequency is higher for older adults compared to young adults and for oVEMPs compared to cVEMPs (31, 30). As clicks have a broad frequency content with maximum energy between about 1 and 4 kHz (32, 33) they are suitable as a general VEMP stimulus. Another limitation of the μVEMP device is that ACS clicks stimulus amplitude cannot be adjusted by the operator (an intentional safety feature). In consequence, a threshold of response, often assessed in SCD or Meniere's patients (34, 35), cannot be evaluated with this device. These limitations have been intentionally imposed for hearing safety reasons, to minimize stimulus generation hardware and for our goal to produce a device that is as easy to use as possible (no frequencies or amplitudes to set).

In conclusion, we have developed and validated a new device to record oVEMPs and cVEMPs in response to sound and vibration. Although further studies and normative data are required for this new device, it is now possible and convenient to assess the function of all ten vestibular end-organs in about 20 min using simple and portable equipment including μVEMP.

## Author contributions

HM developed the device and edited the manuscript. JH developed the device and edited the manuscript. SR recorded comparison data and edited the manuscript. EC recorded and analyzed the data and edited the manuscript.

### Conflict of interest statement

HM is unpaid consultant to GN Otometrics. The remaining authors declare that the research was conducted in the absence of any commercial or financial relationships that could be construed as a potential conflict of interest. The handling Editor declared a shared affiliation, though no other collaboration, with the authors.

## Supplementary material

Figure S1 Schematic block diagram of the μVEMP device.

Figure S2 Cervical VEMP responses from the right ear recorded simultaneously with the μVEMP device (red curves) and the standard CED device (blue curves) from the three healthy subjects in response to ACS clicks and BCV Fz taps. Notice the red curve (μVEMP) is nearly perfectly superimposed with the blue curve (CED).

Figure S3 Ocular VEMP responses from the right ear recorded simultaneously with the μVEMP device (red curves) and the standard CED device (blue curves) from the three healthy subjects in response to ACS clicks and BCV Fz taps. Notice the red curve (μVEMP) is nearly perfectly superimposed with the blue curve (CED).

## Abbreviations

ACS: air-conducted sound
ADCs: Analog to Digital Converters
Amp: Amplitude
BCV: bone-conducted vibration
BVL: bilateral vestibular loss
CED: Cambridge Electronic Design
cVEMPs: Cervical Vestibular Evoked Myogenic Potentials
dB: Decibels
ECG: Electro-Encephalogram
EMG: electromyography
EPr or EP ratio: Evoked Potential ratio
G: gravity
Hz: Hertz
Mm: millimeters
Msec or ms: milliseconds
oVEMPs: Ocular Vestibular Evoked Myogenic Potentials
PP: peak-to-peak
Rc: concordance correlation coefficient
RMS: Root Mean Square
SCD: Superior Canal Dehiscence
SCM: sternocleidomastoid
VEMPs: Vestibular Evoked Myogenic Potentials
vHIT: video-Head Impulse Test
VL: Vestibular loss
μV: microvolt
μVEMP: micro-Vestibular Evoked Myogenic Potentials.
