# Supplementary figures and images for: μVEMP: A Portable Interface to Record Vestibular Evoked Myogenic Potentials (VEMPs) With a Smart Phone or Tablet
Source: Front Neurol. 2018 Jul 5;9:543. doi: 10.3389/fneur.2018.00543 (PMC6042498; doi:10.3389/fneur.2018.00543)

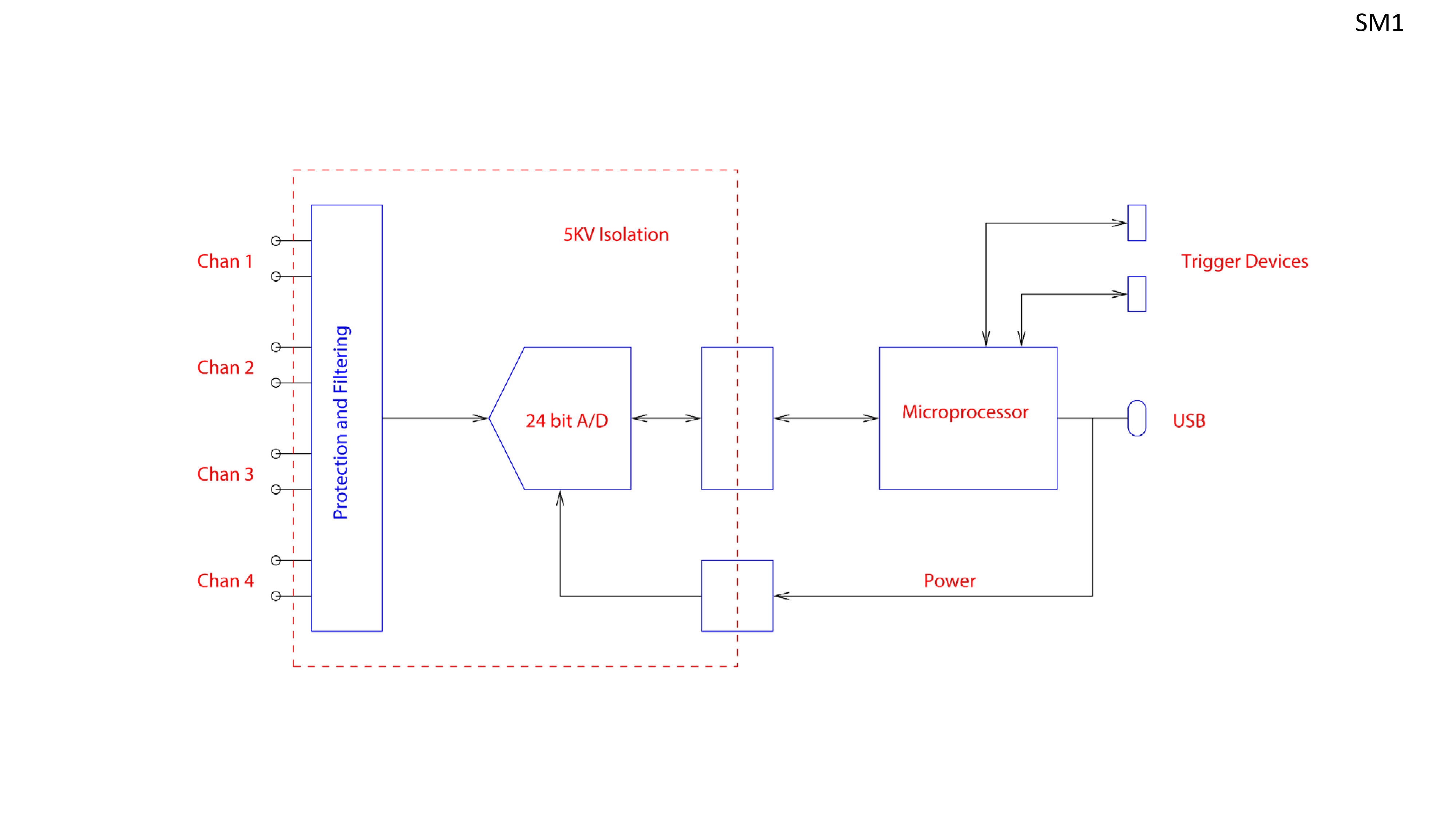

Supplement: Figure S1 — Schematic block diagram of the μVEMP device. [file Image_1.JPEG]

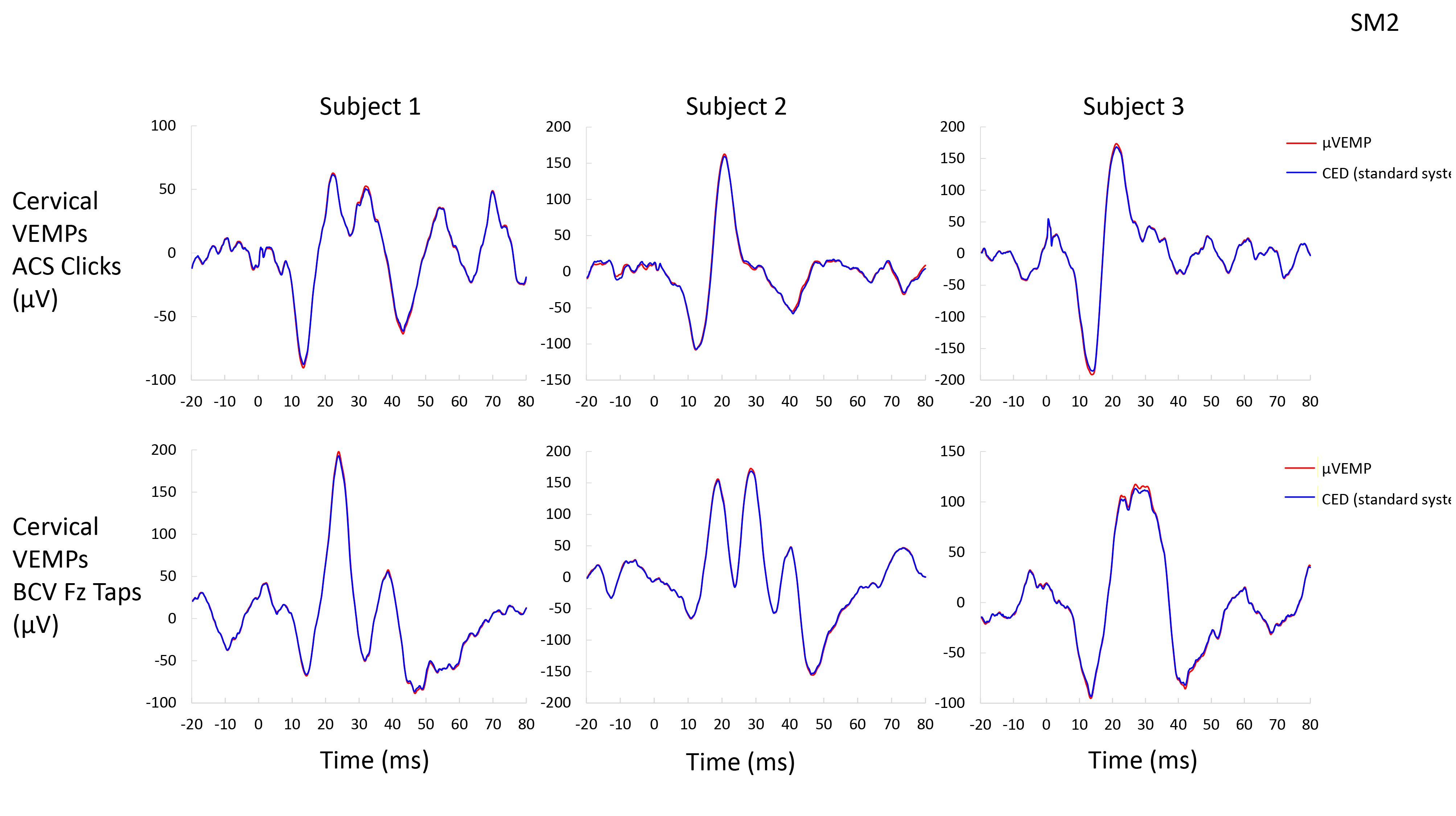

Supplement: Figure S2 — Cervical VEMP responses from the right ear recorded simultaneously with the μVEMP device (red curves) and the standard CED device (blue curves) from the three healthy subjects in response to ACS clicks and BCV Fz taps. Notice the red curve (μVEMP) is nearly perfectly superimposed with the blue curve (CED). [file Image_2.JPEG]

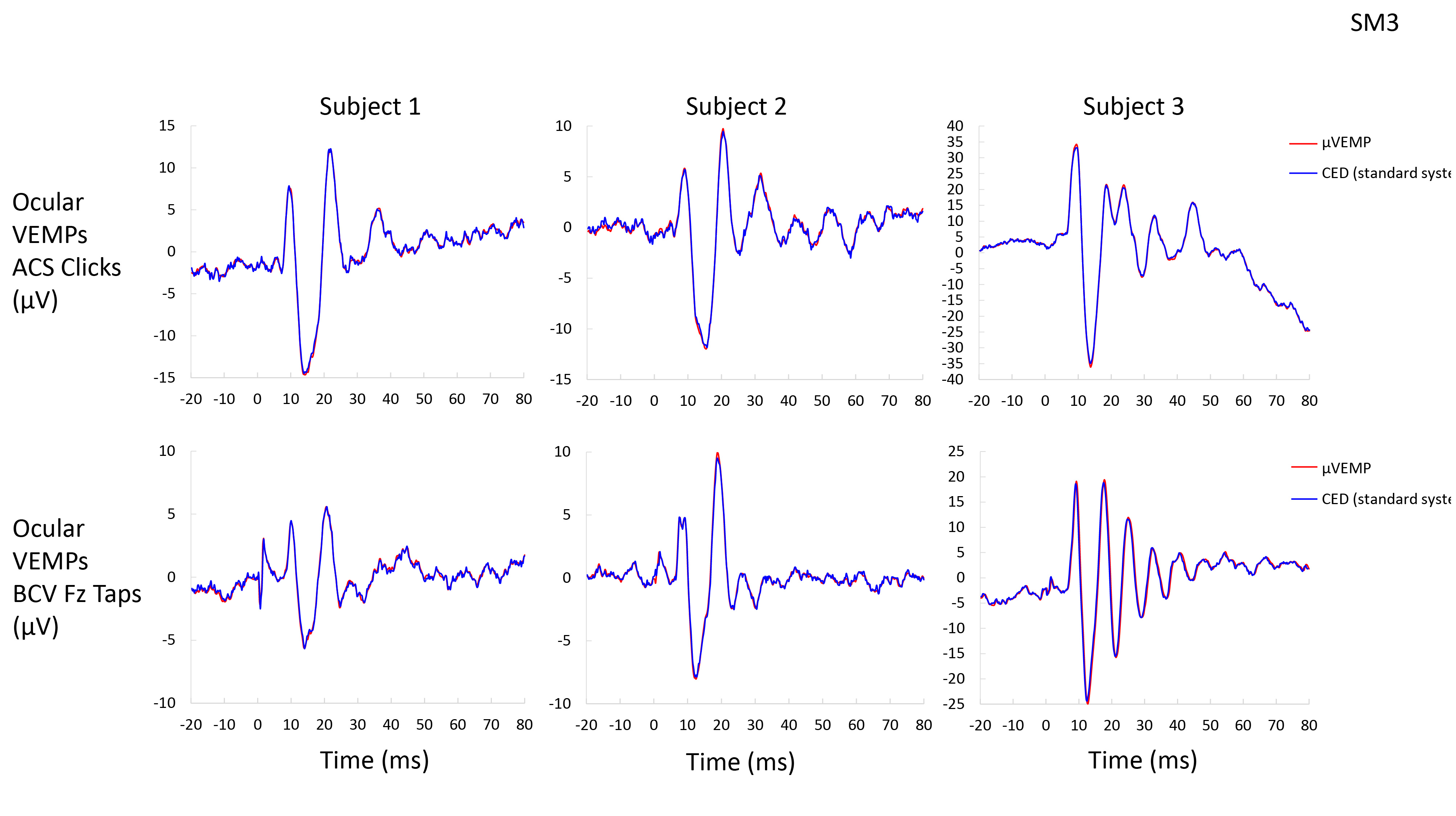

Supplement: Figure S3 — Ocular VEMP responses from the right ear recorded simultaneously with the μVEMP device (red curves) and the standard CED device (blue curves) from the three healthy subjects in response to ACS clicks and BCV Fz taps. Notice the red curve (μVEMP) is nearly perfectly superimposed with the blue curve (CED). [file Image_3.JPEG]
